# Gene Expression Profile for Predicting Survival in Advanced-Stage Serous Ovarian Cancer Across Two Independent Datasets

**DOI:** 10.1371/journal.pone.0009615

**Published:** 2010-03-12

**Authors:** Kosuke Yoshihara, Atsushi Tajima, Tetsuro Yahata, Shoji Kodama, Hiroyuki Fujiwara, Mitsuaki Suzuki, Yoshitaka Onishi, Masayuki Hatae, Kazunobu Sueyoshi, Hisaya Fujiwara, Yoshiki Kudo, Kohei Kotera, Hideaki Masuzaki, Hironori Tashiro, Hidetaka Katabuchi, Ituro Inoue, Kenichi Tanaka

**Affiliations:** 1 Department of Obstetrics and Gynecology, Niigata University Graduate School of Medical and Dental Sciences, Niigata, Japan; 2 Department of Molecular Life Science, Tokai University School of Medicine, Isehara, Japan; 3 Department of Gynecology, Niigata Cancer Center Hospital, Niigata, Japan; 4 Department of Obstetrics and Gynecology, Jichi Medical University, Shimotsuke, Japan; 5 Department of Obstetrics and Gynecology, Kagoshima City Hospital, Kagoshima, Japan; 6 Department of Pathology, Kagoshima City Hospital, Kagoshima, Japan; 7 Department of Obstetrics and Gynecology, Hiroshima University Graduate School of Biomedical Sciences, Hiroshima, Japan; 8 Department of Obstetrics and Gynecology, Nagasaki Municipal Hospital, Nagasaki, Japan; 9 Department of Obstetrics and Gynecology, Nagasaki University Graduate School of Biomedical Sciences, Nagasaki, Japan; 10 Department of Gynecology, Faculty of Medical and Pharmaceutical Sciences, Kumamoto University, Kumamoto, Japan; VU University Medical Center and Center for Neurogenomics and Cognitive Research, The Netherlands

## Abstract

**Background:**

Advanced-stage ovarian cancer patients are generally treated with platinum/taxane-based chemotherapy after primary debulking surgery. However, there is a wide range of outcomes for individual patients. Therefore, the clinicopathological factors alone are insufficient for predicting prognosis. Our aim is to identify a progression-free survival (PFS)-related molecular profile for predicting survival of patients with advanced-stage serous ovarian cancer.

**Methodology/Principal Findings:**

Advanced-stage serous ovarian cancer tissues from 110 Japanese patients who underwent primary surgery and platinum/taxane-based chemotherapy were profiled using oligonucleotide microarrays. We selected 88 PFS-related genes by a univariate Cox model (*p*<0.01) and generated the prognostic index based on 88 PFS-related genes after adjustment of regression coefficients of the respective genes by ridge regression Cox model using 10-fold cross-validation. The prognostic index was independently associated with PFS time compared to other clinical factors in multivariate analysis [hazard ratio (HR), 3.72; 95% confidence interval (CI), 2.66–5.43; *p*<0.0001]. In an external dataset, multivariate analysis revealed that this prognostic index was significantly correlated with PFS time (HR, 1.54; 95% CI, 1.20–1.98; *p* = 0.0008). Furthermore, the correlation between the prognostic index and overall survival time was confirmed in the two independent external datasets (log rank test, *p* = 0.0010 and 0.0008).

**Conclusions/Significance:**

The prognostic ability of our index based on the 88-gene expression profile in ridge regression Cox hazard model was shown to be independent of other clinical factors in predicting cancer prognosis across two distinct datasets. Further study will be necessary to improve predictive accuracy of the prognostic index toward clinical application for evaluation of the risk of recurrence in patients with advanced-stage serous ovarian cancer.

## Introduction

Patients with advanced-stage ovarian cancer generally undergo primary debulking surgery followed by platinum/taxane-based chemotherapy. Although postoperative introduction of taxane drug has improved the 5-year survival rate for advanced-stage ovarian cancer, patients with this cancer have a 5-year survival rate of only 30% [1]–[3]. Clinicopathological characteristics, such as debulking status after primary surgery, are clinically considered important indicators of prognosis [4], [5]. However, recurrence after optimal debulking surgery occurs in some patients, while disease-free status after incomplete surgery is maintained in others. In fact, it has been reported that 34% of patients treated with optimal surgery and platinum-taxane combination chemotherapy for advanced-stage ovarian cancer recur within 12 months [4]. Therefore, these clinicopathological factors alone are insufficient for predicting prognosis and elucidating the pathological mechanisms of disease progression or recurrence. Molecular biology approaches can be used to identify new prognosis-related profiles leading to elucidation of pathological issues of advanced-stage serous ovarian cancer.

Microarray technology has been developing very rapidly, and it has become relatively easy to analyze the expression levels of thousands of genes within cancer cells. Although many studies have reported the associations of gene expression profiles with prognoses in cancer patients [6]–[10], a limited number of such profiles are used in clinical settings. Microarray technology is clinically applied for predicting prognosis in breast cancer patients. MammaPrint ™ (Agendia BV, Amsterdam, the Netherlands) has been already put to practical use for the purpose. Meanwhile, there are no microarray kits for clinical diagnosis and management in patients with ovarian cancer yet.

Three studies have recently reported gene expression profiles that predict overall survival (OS) in ovarian cancer patients using microarray techniques [11]–[13]. These studies use a relative large sample size (n>80) for establishing a survival-related profile in a discovery phase of the experiment and an external independent dataset as the validation set to solve the problem that the number of the genomic variables examined is much larger than that of subjects. Thus, research on the overall survival-related profiles in ovarian cancer patients has progressed, whereas there are no extensive studies based on multicenter validation of gene expression profiles for prediction of disease progression or recurrence in patients with ovarian cancer [14]–[15]. Prediction of the risk of recurrence in patients with advanced-stage ovarian cancer receiving standard treatments (primary surgery+platinum/taxane-based chemotherapy) is more important with respect to optimization of clinical management [16].

We have recently reported that there are high similarities in gene expression between early-stage and a subset of advanced-stage serous ovarian cancer patients that have favorable prognoses, and two molecular subgroups among patients with advanced-stage serous ovarian cancer according to gene expression profiles reflecting tumor progression and prognosis [17]. In this study, we focused on progression-free survival (PFS) time in a larger number of patients only with advanced-stage serous ovarian cancer treated with platinum/taxane-based chemotherapy, and tried to identify PFS-related gene expression profile using a new survival analysis method: ridge regression Cox model [18]. We then assessed the correlation between our PFS-related genes expression profile and survival time in an external independent dataset of advanced-stage serous ovarian cancer.

## Results

### Clinical Characteristics

The clinical characteristics of 110 Japanese patients with advanced-stage serous ovarian cancer are summarized in Table 1. In the discovery set, 93 patients (84.5%) were diagnosed as the International Federation of Gynecology and Obstetrics (FIGO) stage III, and 17 patients (15.5%) as FIGO stage IV [19]. All patients received platinum/taxane-based chemotherapy after primary surgery. The median progression-free and overall survival times were 17 and 31 months, respectively.

**Table 1:** Clinical characteristics of advanced-stage serous ovarian cancer patients.

	Present Dataset (n = 110)	Percentage
Median age, years (range)	58 (23–85)	
Stage		
Stage III	93	84.5
Stage IV	17	15.5
CA125 (IU) (n = 99)	1960±3519	
Optimal Cytoreduction		
Optimal (<1cm)	57	51.8
Not optimal	53	48.2
Grade		
Grade 1	26	23.6
Grade 2	41	37.3
Grade 3	43	39.1
Median survival time, months (range)	31 (1–81)	

On the other hand, we used a part of publicly available microarray data (GSE9891) as an external independent dataset (See Materials and Methods) [20]. The clinical characteristics of 87 patients with advanced-stage serous ovarian cancer in the external dataset are listed in Table S1 [20]. Kaplan-Meier survival analysis showed that there were no significant differences in PFS and OS time between patients of the discovery dataset and those of the external dataset (Figure S1). When we compared clinicopathological characteristics between the discovery set and the external dataset, there were significant differences in frequencies of stage (Table S1). Because grading system adopted in the external dataset was distinct from that in the discovery set [21]–[23], we could not make a simple comparison of malignant grade between the two datasets. Then we examined the association between clinicopathological features and PFS time in patients with advanced-stage serous ovarian cancer of each dataset. Multivariate analysis revealed that only optimal surgery was an independent prognostic factor for PFS in the discovery dataset (Table S2) and that there was marginally significant correlation between debulking status of primary surgery and PFS time in the external dataset (Table S2). Therefore, we planned first to develop a prognostic index based on PFS-related genes in the discovery dataset, secondarily to evaluate the prognostic ability of our index in the external dataset using multivariate analysis, and then thirdly to assess predictive performance of the prognostic index again after the stratification of patients according to the debulking status of primary surgery.

### Identification of PFS-Related Profile

Using Agilent Whole Human Genome Oligo microarray, we generated gene expression data for 110 advanced-stage serous ovarian cancer patients. Then this dataset was used as a discovery set for the identification of PFS-related profile in patients with advanced-stage serous ovarian cancer. To further evaluate the PFS-related profile, we prepared a part of the GSE9891 dataset as an external independent dataset using Affymetrix Human Genome U133 Plus 2.0 Array (See Materials and Methods) [20]. To deal with cross-platform microarray data appropriately, we analyzed only common genes (28304 probes in Agilent platform; 38497 probes in Affymetrix platform) between the two platforms in this study. Of 28304 Agilent probes, 18178 probes with expression levels marked as “Present” in all of the 110 microarray data from the discovery set was further extracted to remove missing and uncertain signals on gene expression, and then the data were per-gene normalized in each dataset by transforming the expression of each gene to a mean of 0 and standard deviation of 1 (Figure S2).

A univariate Cox proportional hazard model showed that expression levels of 97 probes (representing 88 nonredundant genes) were correlated with PFS time (*p*<0.01). In case of multiple-tagged 8 genes (represented by 17 probes), we selected 8 probes (one probe per gene) with the largest sum of the squares of individual expression values for the respective genes as representatives [24]. A total of 88 genes (represented by 88 unique probes) were thereby identified as PFS-related profile. Furthermore, we applied the ridge regression model to estimate optimal regression coefficients (*β*) for 88 genes of the PFS-related profile (Table 2), and calculated the prognostic index for each sample using equation (1) as reported previously [18]. The 88-gene prognostic indices obtained were in the range of -5.09 to 4.14 (median, 0.11), and the frequency distribution of the indices among 110 patients was unimodal.

**Table 2:** Eighty-eight genes composing the progression-free survival-related profile.

GenBank Acc.	GeneSymbol	Cytoband	*β*_ridge_ a	Description
NM_001123	*ADK*	10q22.2	0.006	adenosine kinase
NM_006408	*AGR2*	7p21.1	0.128	anterior gradient homolog 2 (Xenopus laevis)
NM_080429	*AQP10*	1q21.3	−0.162	aquaporin 10
NM_001040118	*ARAP1*	11q13.4	0.141	ArfGAP with RhoGAP domain, ankyrin repeat and PH domain 1
NM_006420	*ARFGEF2*	20q13.13	0.032	ADP-ribosylation factor guanine nucleotide-exchange factor 2 (brefeldin A-inhibited)
NM_181575	*AUP1*	2p13.1	0.129	ancient ubiquitous protein 1
NM_004776	*B4GALT5*	20q13.13	0.215	UDP-Gal:betaGlcNAc beta 1,4- galactosyltransferase, polypeptide 5
NM_138639	*BCL2L12*	19q13.33	−0.189	BCL2-like 12 (proline rich)
NM_020643	*C11orf16*	11p15.4	0.221	chromosome 11 open reading frame 16
NM_145061	*C13orf3*	13q12.11	−0.107	chromosome 13 open reading frame 3
NM_024032	*C17orf53*	17q21.31	−0.184	chromosome 17 open reading frame 53
NM_001144956	*C1orf230*	1q21.3	0.012	chromosome 1 open reading frame 230
NM_022106	*C20orf177*	20q13.33	0.167	chromosome 20 open reading frame 177
NM_000715	*C4BPA*	1q32.2	−0.505	complement component 4 binding protein, alpha
NM_012337	*CCDC19*	1q23.2	−0.162	coiled-coil domain containing 19
NM_015603	*CCDC9*	19q13.32	0.263	coiled-coil domain containing 9
NM_005408	*CCL13*	17q12	−0.228	chemokine (C-C motif) ligand 13
NM_001252	*CD70*	19p13.3	−0.204	CD70 molecule
NM_078481	*CD97*	19p13.12	−0.137	CD97 molecule
NM_006383	*CIB2*	15q25.1	0.359	calcium and integrin binding family member 2
NM_182848	*CLDN10*	13q32.1	−0.292	claudin 10
NM_001316	*CSE1L*	20q13.13	−0.220	CSE1 chromosome segregation 1-like (yeast)
NM_024295	*DERL1*	8q24.13	0.007	Der1-like domain family, member 1
NM_001042517	*DIAPH3*	13q21.2	0.022	diaphanous homolog 3 (Drosophila)
NM_021120	*DLG3*	Xq13.1	−0.039	discs, large homolog 3 (Drosophila)
NM_020877	*DNAH2*	17p13.1	−0.378	dynein, axonemal, heavy chain 2
NM_018897	*DNAH7*	2q32.3	0.226	dynein, axonemal, heavy chain 7
NM_001394	*DUSP4*	8p21.1	0.007	dual specificity phosphatase 4
NM_004091	*E2F2*	1p36.12	0.220	E2F transcription factor 2
NM_006795	*EHD1*	11q13.1	0.248	EH-domain containing 1
NM_020819	*FAM135A*	6q13	0.142	family with sequence similarity 135, member A
NM_032181	*FAM176A*	2p12	−0.096	family with sequence similarity 176, member A
NM_015687	*FILIP1*	6q14.1	−0.188	filamin A interacting protein 1
NM_021784	*FOXA2*	20p11.21	0.184	forkhead box A2
NM_001454	*FOXJ1*	17q25.1	−0.344	forkhead box J1
NM_000819	*GART*	21q22.11	0.140	phosphoribosylglycinamide formyltransferase, phosphoribosylglycinamide synthetase, phosphoribosylaminoimidazole synthetase
NM_178172	*GPIHBP1*	8q24.3	0.147	glycosylphosphatidylinositol anchored high density lipoprotein binding protein 1
NM_000189	*HK2*	2p13.1	−0.087	hexokinase 2
NM_002118	*HLA-DMB*	6p21.32	−0.288	major histocompatibility complex, class II, DM beta
NM_022465	*IKZF4*	12q13.2	−0.092	IKAROS family zinc finger 4 (Eos)
NM_016584	*IL23A*	12q13.2	0.493	interleukin 23, alpha subunit p19
NM_006801	*KDELR1*	19q13.32	−0.001	KDEL (Lys-Asp-Glu-Leu) endoplasmic reticulum protein retention receptor 1
NM_014895	*KIAA1009*	6q14.3	−0.150	KIAA1009
NM_017527	*LY6K*	8q24.3	0.226	lymphocyte antigen 6 complex, locus K
NM_005906	*MAK*	6p24.2	0.271	male germ cell-associated kinase
NM_024871	*MAP6D1*	3q27.1	−0.038	MAP6 domain containing 1
NM_031417	*MARK4*	19q13.32	0.040	MAP/microtubule affinity-regulating kinase 4
NM_024298	*MBOAT7*	19q13.42	−0.058	membrane bound O-acyltransferase domain containing 7
NM_002421	*MMP1*	11q22.2	−0.336	matrix metallopeptidase 1 (interstitial collagenase)
NM_181526	*MYL9*	20q11.23	0.058	myosin, light chain 9, regulatory
NM_032344	*NUDT22*	11q13.1	0.198	nudix (nucleoside diphosphate linked moiety X)-type motif 22
NM_007224	*NXPH4*	12q13.3	−0.310	neurexophilin 4
NM_015311	*OBSL1*	2q35	−0.045	obscurin-like 1
NM_014982	*PCNX*	14q24.2	−0.098	pecanex homolog (Drosophila)
NM_014317	*PDSS1*	10p12.1	0.001	prenyl (decaprenyl) diphosphate synthase, subunit 1
NM_024420	*PLA2G4A*	1q31.1	0.107	phospholipase A2, group IVA (cytosolic, calcium-dependent)
NM_016341	*PLCE1*	10q23.33	0.029	phospholipase C, epsilon 1
NM_001031745	*RIBC1*	Xp11.22	0.209	RIB43A domain with coiled-coils 1
NM_015653	*RIBC2*	22q13.31	0.053	RIB43A domain with coiled-coils 2
NM_006987	*RPH3AL*	17p13.3	−0.043	rabphilin 3A-like (without C2 domains)
NM_001025070	*RPS14*	5q33.1	0.013	ribosomal protein S14
NM_152732	*RSPH9*	6p21.1	−0.102	radial spoke head 9 homolog (Chlamydomonas)
NM_014433	*RTDR1*	22q11.22	0.034	rhabdoid tumor deletion region gene 1
NM_005500	*SAE1*	19q13.32	0.038	SUMO1 activating enzyme subunit 1
NM_020150	*SAR1A*	10q22.1	0.277	SAR1 homolog A (S. cerevisiae)
NM_031469	*SH3BGRL2*	6q14.1	−0.281	SH3 domain binding glutamic acid-rich protein like 2
NM_003951	*SLC25A14*	Xq25	−0.344	solute carrier family 25 (mitochondrial carrier, brain), member 14
NM_014585	*SLC40A1*	2q32.2	0.065	solute carrier family 40 (iron-regulated transporter), member 1
NM_052910	*SLITRK1*	13q31.1	−0.314	SLIT and NTRK-like family, member 1
NM_172312	*SPAG8*	9p13.3	−0.123	sperm associated antigen 8
NM_145263	*SPATA18*	4q12	0.041	spermatogenesis associated 18 homolog (rat)
NM_006100	*ST3GAL6*	3q12.1	−0.192	ST3 beta-galactoside alpha-2,3-sialyltransferase 6
NM_018414	*ST6GALNAC1*	17q25.1	−0.175	ST6 (alpha-N-acetyl-neuraminyl-2,3-beta-galactosyl-1,3) -N-acetylgalactosaminide alpha-2,6-sialyltransferase 1
NM_032872	*SYTL1*	1p36.11	−0.084	synaptotagmin-like 1
NM_014466	*TEKT2*	1p34.3	−0.226	tektin 2 (testicular)
NM_005424	*TIE1*	1p34.2	0.250	tyrosine kinase with immunoglobulin-like and EGF-like domains 1
NM_198276	*TMEM17*	2p15	0.025	transmembrane protein 17
NM_199203	*TMEM189 -UBE2V1*	20q13.13	0.174	TMEM189-UBE2V1 readthrough transcript
NM_033550	*TP53RK*	20q13.12	0.054	TP53 regulating kinase
NM_139075	*TPCN2*	11q13.2	0.034	two pore segment channel 2
NM_018430	*TSNAXIP1*	16q22.1	0.170	translin-associated factor X interacting protein 1
NM_014023	*WDR37*	10p15.3	0.296	WD repeat domain 37
NM_018053	*XKR8*	1p35.3	0.106	XK, Kell blood group complex subunit-related family, member 8
NM_015896	*ZMYND10*	3p21.31	0.052	zinc finger, MYND-type containing 10
NM_005773	*ZNF256*	19q13.43	0.048	zinc finger protein 256
NM_024691	*ZNF419*	19q13.43	−0.042	zinc finger protein 419
NM_021089	*ZNF8*	19q13.43	0.093	zinc finger protein 8
NM_017975	*ZWILCH*	15q22.31	−0.074	Zwilch, kinetochore associated, homolog (Drosophila)

a A regression coefficient of each gene in ridge regression extension of multivariate Cox hazard model.

**Figure 1:**
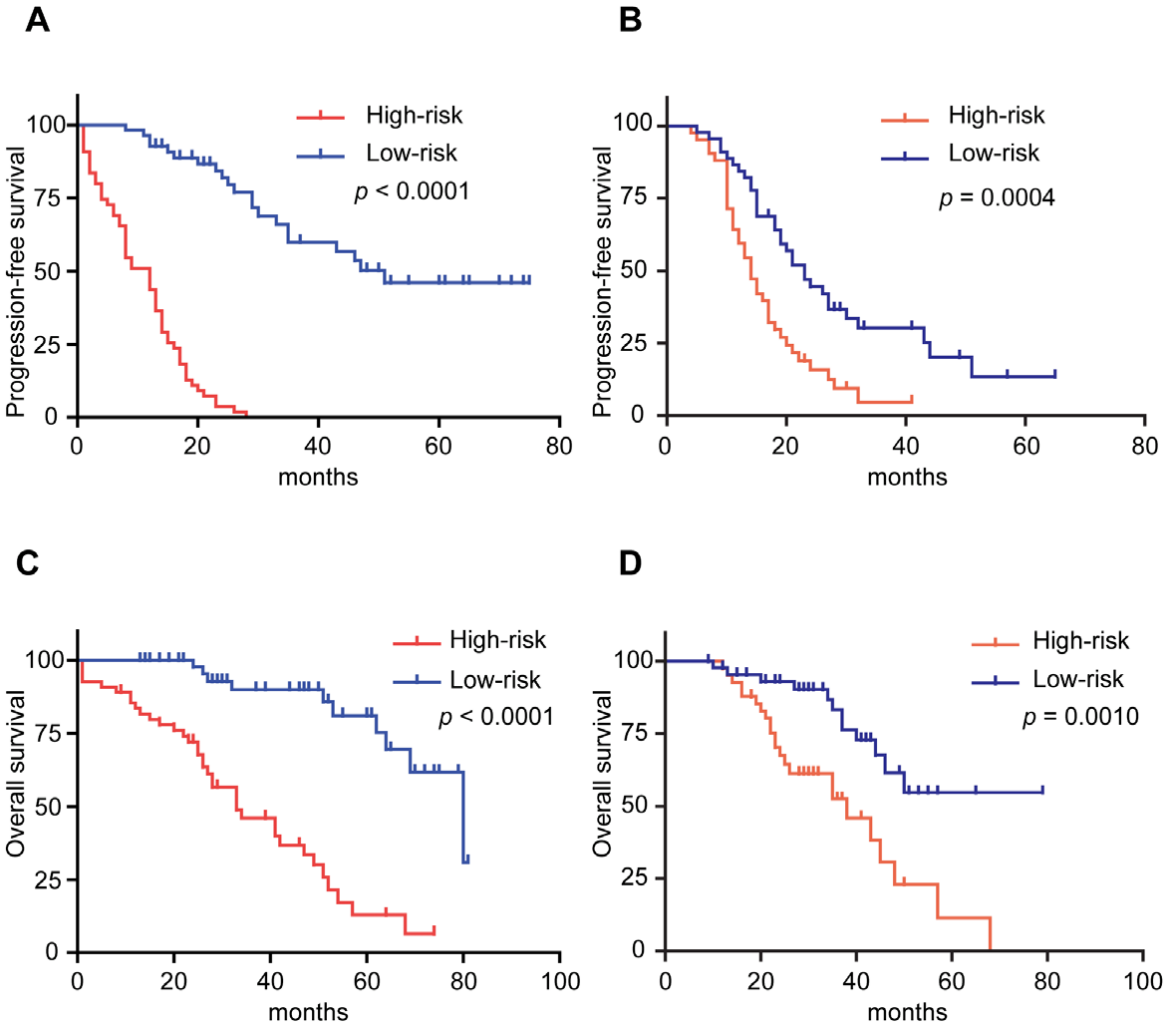
Prediction of prognosis in high-risk and low-risk patients based on the prognostic index. High-risk patients had significantly short progression-free survival times compared to low-risk patients (A) in the discovery set (log rank test, *p*<0.0001) and (B) in the external set (log rank test, *p* = 0.0004). Similarly, high-risk patients had significantly shorter overall survival times compared to low-risk patients (C) in the discovery set (log rank test, *p*<0.0001) and (D) in the external set (log rank test, *p* = 0.0010).

To assess the prognostic index as a categorical variable, we attempted to divide this dataset into two groups based on median prognostic index of 0.11 [9]. Patients were assigned to the “high-risk” group if their prognostic index was greater than or equal to the median value, whereas “low-risk” group was composed of cases with the prognostic indices that were less than the median. As shown in Figure 1A, patients with high-risk prognostic indices had shorter median PFS times than those belonging to low-risk group (median PFS, 12 months vs. 51 months; log rank test, *p*<0.0001).

We then performed univariate and multivariate Cox proportional hazard analyses to prove that the 88-gene prognostic index was an independent prognostic factor (Table 3). A univariate Cox's proportional hazard analysis showed that the prognostic index, stage, optimal surgery, and histological grade were correlated with PFS (*p*<0.0001, *p* = 0.022, *p*<0.0001 and *p* = 0.016, respectively). Moreover, a multivariate analysis showed that the prognostic index was most significantly associated with PFS time [hazard ratio (HR), 3.80; 95% confidence interval (CI), 2.68–5.61; *p*<0.0001].

**Table 3:** Univariate and multivariate Cox's proportional hazard model analysis of prognostic factors for progression-free survival.

	Univariate analysis		Multivariate analysis	
Prognostic factor	Hazard ratio (95%CI*)	*p*-value	Hazard ratio (95%CI)	*p*-value
**A) Present study (n = 110)**				
** Age**	0.99 (0.97–1.01)	0.41	1.00 (0.99–1.02)	0.68
** Stage IV (vs Stage III)**	1.40 (1.05–1.81)	0.022	0.93 (0.69–1.24)	0.65
** Optimal Surgery (vs not optimal)**	0.57 (0.45–0.72)	<0.0001	0.73 (0.56–0.94)	0.016
** Grade**				
** Grade2 (vs Grade1)**	1.21 (0.89–1.67)	0.23	1.08 (0.78–1.50)	0.66
** Grade3 (vs Grade1)**	1.44 (1.07–1.98)	0.016	1.34 (0.98–1.88)	0.065
** Prognostic Index**				
** High (vs Low)**	3.95 (2.85–5.74)	<0.0001	3.80 (2.68–5.61)	<0.0001
**B) Tothill's dataset ** [**20**] ** (n = 87)**				
** Age**	1.01 (0.98–1.03)	0.61	1.00 (0.98–1.03)	0.82
** Stage IV (vs Stage III)**	1.26 (0.51–2.28)	0.55	0.83 (0.33–1.55)	0.60
** Optimal Surgery (vs not optimal)**	0.78 (0.62–0.99)	0.049	0.76 (0.60–0.98)	0.035
** Prognostic Index**				
** High (vs Low)**	1.62 (1.26–2.09)	0.0001	1.64 (1.27–2.13)	0.0001

*CI denotes confidence interval.

### Validation by Quantitative Real-Time RT-PCR

**Figure 2:**
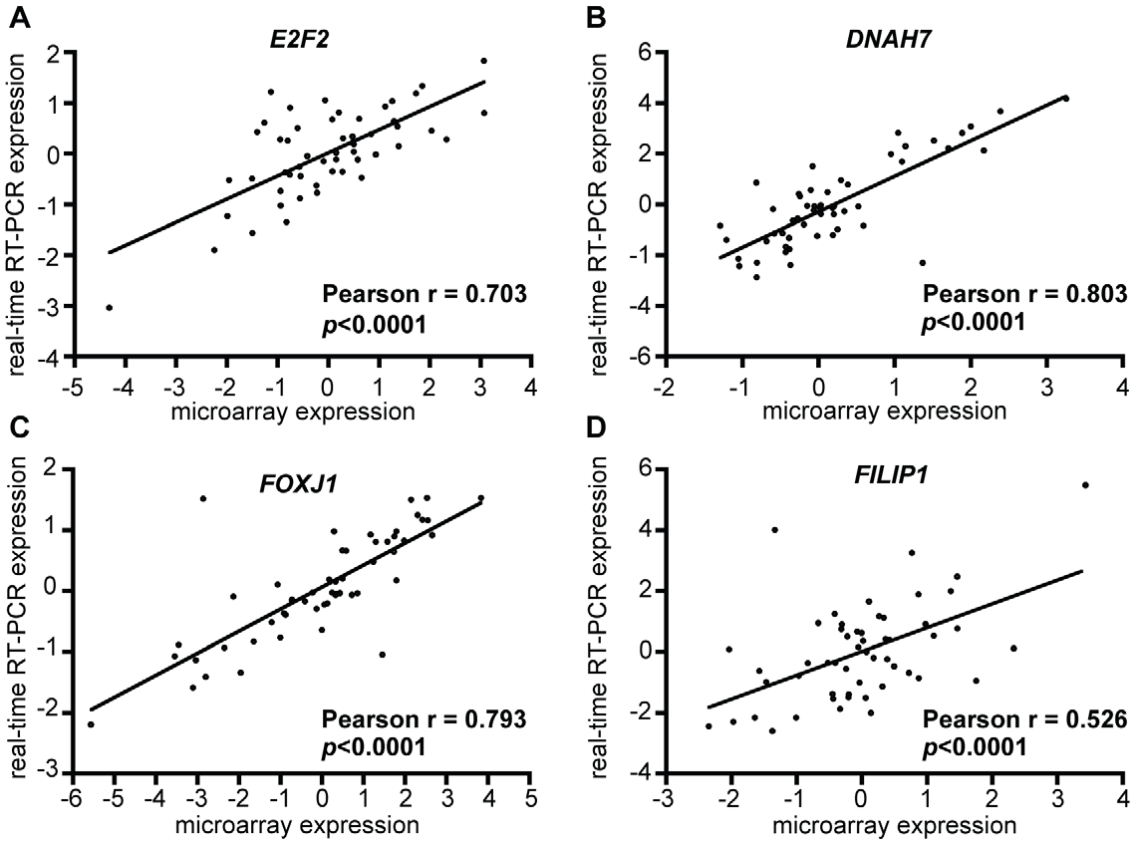
Validation of microarray expression data using quantitative real-time reverse transcript polymerase chain reaction (RT-PCR) analysis. There were significant correlations between microarray expression and real-time RT-PCR expression in (A) *E2F2*, (B) *DNAH7*, (C) *FOXJ1*, and (D) *FILIP1*.

To validate the microarray expression data, we performed quantitative real-time RT-PCR for a subset of the discovery dataset (53 samples). The four genes, *E2F2*, *FOXJ1*, *DNAH7*, and *FILIP1*, were randomly selected for this purpose. There were significant correlations between microarray expression data and real-time RT-PCR expression data (Figure 2). In spite of the smaller sample size, we confirmed a significant association between PFS time and each of the real-time RT-PCR data for the four genes in the univariate Cox hazard model (data not shown).

### Appling PFS-Related Profile to the External Dataset

We translated the 88 prognostic genes with Agilent Probe IDs to Affymetrix 196 probes using a translation function in GeneSpring GX 10 and evaluated the present PFS-related profile in the external dataset (Figure S2). We calculated the prognostic index for each sample in the external dataset by the weighted sum of the expression values of 88 PFS-related genes according to the equation (1), in which the ridge regression coefficients (*β*) identified in the discovery set were used as weights for the respective genes (See Materials and Methods). We obtained prognostic indices ranging from -5.37 to 4.56 in the external dataset. The frequency distribution of the prognostic indices was not statistically different from that in the discovery set by Kolmogorov Smirnov test (*p*>0.05).

When we divided the external dataset into two subgroups by the median prognostic index (0.11) in the discovery set, a significant correlation was observed between risk classification and PFS (log rank test, *p* = 0.0004) (Figure 1B). In univariate analysis of the external data, the estimated prognosis index and optimal surgery were correlated with PFS time (*p* = 0.0001 and 0.049, respectively) (Table 3). Multivariate analysis showed that prognostic index was an independent prognostic factor for PFS time (HR, 1.64; 95% CI, 1.27–2.13, *p* = 0.0001).

### Assessment of Our Prognostic Index

To assess the sensitivity and specificity of our prognostic index, we used ROC curves for the index. An area under ROC curve of 0.5 (indicated by diagonal dotted lines in Figure S3) represents equality between true positive and false positive test results. The extent to which the ROC curve departs from the diagonal line to left and top axes is a measure of the effectiveness of the 88-gene prognostic index in the prediction of clinical outcome. The area under the ROC curves to distinguish early-relapse patients with less than 18 months of PFS times from late-relapse patients was 0.959 and 0.674 in the discovery set and the external dataset, respectively (Figure S3). When we used median value of prognostic index in the discovery set as the cut-off, the sensitivity and specificity were 88.9% and 85.7% in discovery dataset and 64.4% and 69.2% in the external dataset.

**Figure 3:**
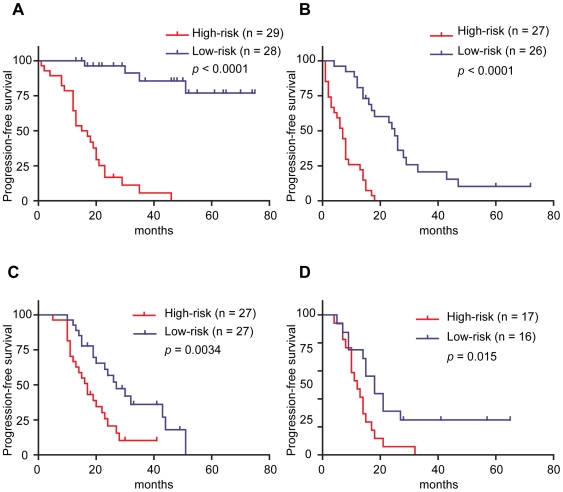
Prediction of prognosis in high-risk and low-risk patients based on the prognostic index after the stratification of patients according to the status of debulking surgery. High-risk patients had significantly short progression-free survival times compared to low-risk patients (A) in optimal (log rank test, *p*<0.0001) and (B) suboptimal group of discovery dataset (log rank test, *p*<0.0001). Similarly, high-risk patients had significantly shorter overall survival times compared to low-risk patients (C) in optimal (log rank test, *p* = 0.0034) and (D) suboptimal group of the external dataset (log rank test, *p* = 0.015).

We performed survival analysis after the stratification of patients according to the status of debulking surgery which was an independent prognostic factor in multivariate analysis of the discovery dataset (Table 3). We divided patients into two groups (“optimal group” and “suboptimal group”) in each of the discovery and external datasets, and assigned each patient to “high-risk” or “low-risk” based on the median value of the current prognostic index in each stratum according to the debulking status. Kaplan-Meier survival analysis showed that high-risk patients had significant shorter PFS time than low-risk patients in each of the four strata from the two datasets (Figure 3) as follows: optimal group (*p*<0.0001) and suboptimal group (*p*<0.0001) in our dataset; optimal group (*p* = 0.0034) and suboptimal group (*p* = 0.015) in the external dataset. This stratified analysis also indicated that the prognostic index was associated with PFS time independently of the debulking status.

### Correlation between This Prognostic Index and Overall Survival

Overall survival is another important endpoint in patients with advance-stage ovarian cancers, and hence we further examined if the present 88-gene prognostic index could be extended to use for predicting the overall survival of patients. To evaluate correlation between this prognostic index and overall survival time, we performed Kaplan-Meier survival curve analysis. Patients with high-risk prognostic indices had shorter overall survival times than the low-risk patients in the two datasets (log rank test, *p*<0.0001 and *p* = 0.0010, respectively) (Figure 1C, D). Furthermore, the prognostic index was significantly associated with overall survival time in both the discovery set and the external dataset in multivariate analysis (Table 4).

**Table 4:** Univariate and multivariate Cox's proportional hazard model analysis of prognostic factors for overall survival.

	Univariate analysis		Multivariate analysis	
Prognostic factor	Hazard ratio (95%CI*)	*p*-value	Hazard ratio (95%CI)	*p*-value
**A) Present study (n = 110)**				
** Age**	1.01 (0.98–1.03)	0.56	-	-
** Stage IV (vs Stage III)**	1.14 (0.78–1.59)	0.49	0.75 (0.50–1.08)	0.12
** Optimal Surgery (vs not optimal)**	0.69 (0.50–0.92)	0.012	0.98 (0.70–1.35)	0.90
** Grade**				
** Grade2 (vs Grade1)**	1.30 (0.85–2.09)	0.23	1.23 (0.80–2.01)	0.35
** Grade3 (vs Grade1)**	1.68 (1.12–2.68)	0.012	1.83 (1.18–3.02)	0.0065
** Prognostic Index**				
** High (vs Low)**	2.72 (1.91–4.08)	<0.0001	2.99 (2.02–4.65)	<0.0001
**B) Tothill's dataset ** [**20**] ** (n = 87)**				
** Age**	1.01 (0.97–1.05)	0.73	1.00 (0.97–1.04)	0.88
** Stage IV (vs Stage III)**	2.13 (0.85–3.95)	0.093	1.60 (0.62–3.21)	0.28
** Optimal Surgery (vs not optimal)**	0.89 (0.62–1.23)	0.42	0.94 (0.66–1.37)	0.74
** Prognostic Index**				
** High (vs Low)**	1.76 (1.24–2.55)	0.0013	1.71 (1.20–2.49)	0.0029

*CI denotes confidence interval.

In addition, we examined the predictive ability of our prognostic index in publicly available Dressman's dataset [25], in which patients were longer followed-up (median overall survival, 31 months; range, 1–185 months). Dressman's dataset [25] was composed of 119 advanced-stage serous ovarian cancer patients treated with platinum-based chemotherapy (including non-taxane chemotherapy). Because their data were generated by a different platform with the foregoing two datasets, 75% of 88 PFS-related genes were translated for survival prediction in this dataset. When we divided Dressman's dataset [25] into two subgroups by the median prognostic index in discovery dataset, a significant association was observed between risk classification and overall survival (log rank test, *p* = 0.0008) (Figure S4). Its prognostic index was significantly correlated with overall survival time in multivariate analysis (HR, 1.51; 95% CI, 1.19–1.93, *p* = 0.0008).

### Characterization of PFS-Related Profile

**Figure 4:**
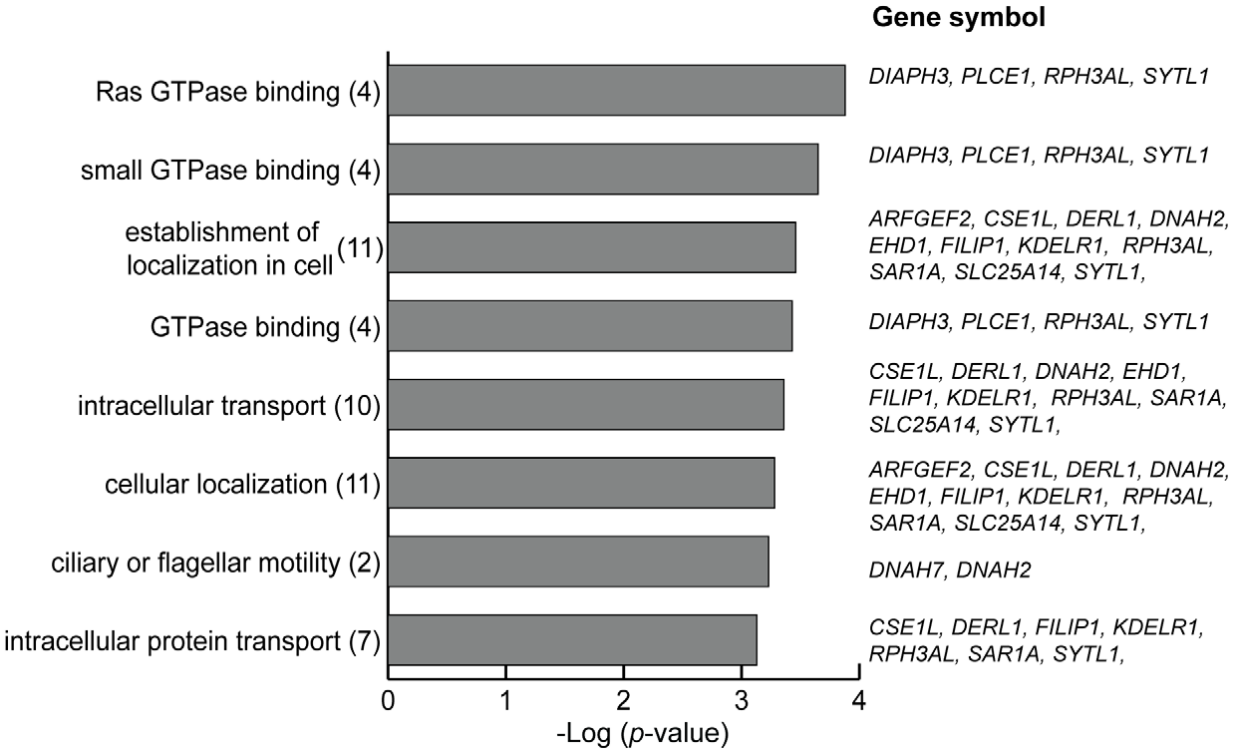
Biological characteristics of 88 progression-free survival-related genes. Significantly over-represented 8 gene ontology (GO) categories in GO-based profiling of 88 genes after multiple testing correction of the Benjamini–Hochberg false discovery rate method (FDR *q*-value<0.10). Over-represented GO categories were identified using all genes on Agilent platform as a background set of genes for the determining *p*-values. The actual number of the PFS-related genes involved in each category is given in parentheses.

We conducted GO analysis to understand the biological characteristics of 88 PFS-related genes. To characterize the gene list based on GO classification on ‘biological process’, ‘molecular function’, and ‘cellular component’, we examined which categories were highly associated with the 88 genes. After multiple testing corrections using the FDR method [26], 8 categories were significantly overrepresented (FDR *q*-value<0.10) (Figure 4). In the 88 PFS-related genes, genes involved in GTPase binding (GO17016, GO31267 and GO51020), cellular localization (GO51649 and GO51641), intracellular transport (GO46907 and GO6886), and/or ciliary or flagellar motility (GO1539) were notably enriched. We investigated similarities in overrepresented GO categories between our 88 PFS-related genes and the previously reported gene expression profiles which were correlated to prognosis in ovarian cancer [11], [13]. However, we could not identify common GO categories between our profile and the previously reported profiles (data not shown).

We further used IPA software to analyze 88 PFS-related genes from the viewpoint of molecular interaction or pathway. Top three significant networks (score>25) are shown in Figures S5-7. The network 1 included 15 of the 88 prognostic genes, and was significantly associated with IPA-defined several networks: cell death, neurological disease, and cellular assembly and organization (Figure S5). Fourteen prognostic genes were included in the network 2, which was defined as networks related to cancer, cell morphology, and renal and urological disease (Figure S6). The network 3 displayed significant interactions and interrelations between genes involved in cell-to-cell signaling and interaction, hematological system development and function, and immune cell trafficking (Figure S7). In the 88 genes, we found several genes interacting with *SRC* or *MYC* (Figure S6), each of which was reported as a representative gene in oncogenic pathways of ovarian cancer [25], [27].

## Discussion

In this study, we identified the prognostic index for predicting PFS time in patients with advanced-stage serous ovarian cancer treated with platinum/taxane-based adjuvant chemotherapy across two types of microarray expression data from the present discovery set and publicly available external set by using the ridge regression Cox model. The significant correlation between our prognostic index and OS time was also indicated in the two independent datasets.

In expression microarray analysis, there is a so-called “curse of dimensionality” problem that the number of genes is much larger than the number of samples. To improve the reliability of a gene expression-based prognostic model, it is necessary to avoid overfitting to the dataset, and to confirm the reproducibility of the predictive ability in external independent datasets [28]. Until now, several bioinformatics approaches have been proposed to establish a model for survival prediction using microarray data [18], [29]. Bøvelstad *et al*. [18] recently examined the prediction performance of the following seven methods: univariate selection, forward stepwise selection, principal components regression, supervised principal components regression, partial least squares regression, ridge regression and the lasso using three microarray datasets [Dutch breast cancer data (n = 295), diffuse large B-cell lymphoma data (n = 240), and Norway/Stanford breast cancer data (n = 115)] [7], [30]–[32]. They concluded that the univariate Cox model alone was insufficient for predicting survival and that the ridge regression Cox model demonstrated the best performance in three datasets. Therefore, we used univariate Cox model only for selecting genes related to PFS time, and adjusted the regression coefficients by the ridge regression Cox model in order to increase the predictive performance of the prognostic index in our dataset.

The current study is intended to identify gene expression profile with a superior ability to predict prognosis than other clinicopathological factors. The stratification of patients with ovarian cancer according to clinicopathological prognostic factors is one of important analysis methods for the identification of highly accurate prognostic index [11]. After we stratified patients according to grade, FIGO stage, and status of debulking surgery, we investigated gene expression profile for predicting PFS time in stage III grade 2/3 serous ovarian cancer patients received optimal surgery or suboptimal surgery. However, we could find poorer predictive performance of the prognostic indices from the stratified analyses than that from the non-stratified analysis (Table S3). Besides the reduction of sample size in the discovery and external datasets after the stratification, a variety in clinical features and grading systems between the two datasets (Table S1) might influence the results from these stratified analyses. This is the main reason why we planned to identify prognostic index based on PFS-related genes in 110 advanced-stage serous ovarian cancers and then evaluate the significance of the prognostic index using multivariate analysis including grade, stage, and status of debulking surgery.

Although we enrolled ovarian cancer patients screened carefully by the following three categories: advanced-stage, histological serous-type, and platinum/taxane-based chemotherapy after primary surgery, we established no inclusion or exclusion criterion of histological grade for the enrollment as well as Crijns and colleagues did [12]. This is because a standard system for grading ovarian carcinomas is still under construction in the world, although several grading systems have been proposed for epithelial ovarian cancer [21]–[23], [33], [34]. According to the three criteria above, we recruited 110 Japanese ovarian cancer patients as a discovery set for the PFS analysis. The prognostic index for each patient was simply calculated by the ridge-regression-weighted sum of 88-gene expression values, and the prognostic power of our index was assessed using Tothill's dataset [20]. Further, subsequent stratified analysis according to debulking status, which was an independent prognostic factor in multivariate analysis of the discovery dataset, indicated that our prognostic index was associated with PFS time independently of the debulking status. However, the sensitivity and specificity of the prognostic index for discriminating between early- and late-relapse patients were lower in Tothill's dataset than those in the discovery set. This might be caused by different backgrounds in respects of ethnicity or microarray platform. Although the differences in gene expression of cancer tissues among ethnicities have not been reported previously, several studies indicate that the proportions of clear cell and endometrioid histological types in epithelial ovarian cancer in Asian population are higher than those in non-Asian populations [35], [36]. Recent genome-wide association study has identified a single nucleotide polymorphism at 9p22 associated with ovarian cancer risk in subjects with European ancestry but not in non-European descendants [37]. This type of differences between studies could be also attributed to genetic as well as environmental factors. In addition, we cannot rule out the possibility that the present PFS-associated classifiers with ridge-regression-based weights still have insufficient generalization properties on the external dataset due to the problem of overfitting. Therefore, we will reconsider these important issues such as between-study differences in ethnicities and microarray platforms and the overfitting problem using a larger number of microarray data from advanced-stage serous ovarian cancer patients in order to obtain better classifiers for the prediction of prognosis. And to improve the accuracy of prognostic index, development of prognostic index after the stratification of patients will be a research agenda for further study.

Interestingly, the present 88-gene prognostic index for prediction of PFS time was also significantly associated with overall survival time in both our dataset and Tothill's dataset [20]. Moreover, we examined the predictive ability of our prognostic index in Dressman's dataset [25] since patients in their dataset received longer-term follow-up than those in the above two datasets. Although Dressman's dataset (n = 119) [25] included 34 patients treated with platinum/cyclophospamide chemotherapy and 3 with single-agent platinum, the significance of this prognostic index for overall survival was still statistically supported in the longer followed-up dataset. As treatments for recurrent ovarian cancer patients remain an open area of investigation aiming to lead to survival benefit [38], our prognostic index for patient with advanced-stage serous ovarian cancer displays a potential to predict not only PFS time but also overall survival time. In the future, we may apply the prognostic indices to estimation of risk of recurrence for serous ovarian cancer patients and select a novel treatment such as dose-dense chemotherapy [39] or molecular-targeted agent for the purpose of improving prognosis of high-risk patients.

There are small number of genes overlapped between our 88 PFS-related profile and previously reported expression-profiles that were related to prognosis or sensitivity of platinum/taxane-based chemotherapy [11]–[15], [40], [41]. Konstantinopoulos *et al*. [6] have discussed that these discrepancies might be related to the use of different microarray platforms with different normalization methods and different degree of contamination by noncancerous cells in a tumor sample, as well as differences in the patient populations under study. Nevertheless, several survival-associated genes such as *E2F2* and *HLA-DMB* [42], [43] are included in 88 PFS-related genes. Reimer *et al*. [42] have reported that *E2F2* is associated with grade 3 ovarian tumors and residual disease (more than 2cm in diameter) after initial surgery, and that low *E2F2* expression is significantly associated with favorable disease-free and overall survival in epithelial ovarian cancer. Callahan *et al*. [43] have recently reported that the high expression of HLA-DMB in ovarian cancer cells is correlated with increased numbers of tumor-infiltrating CD8-positive T lymphocytes, and with good prognosis in advanced-stage high-grade serous ovarian cancer.

We performed GO analysis and IPA to assess biological characteristics of PFS-related genes. GO analysis revealed the significant associations of GTPase binding, intracellular transport, and ciliary or flagellar motility with PFS (Figure 4). *PLCE1* belongs to the GTPase binding category and activates MAP kinase or ERK as shown in IPA network 3 (Figure S7). In particular, previous report indicates that *PLCE1* activates the small G protein Ras/MAP kinase signaling [44], which is one of important pathways associated with cell growth and differentiation. Intriguingly, *CSE1L* included in the intracellular transport category is involved in the regulation of multiple cellular mechanisms, proliferation, and apoptosis [45]. Tanaka *et al*. [46] have reported that *CSE1L* is associated with regulated expression of p53 target genes, and that downregulation of *CSE1L* protects cancer cell from DNA damage-induced apoptosis. *DNAH2* and *DNAH7* are components of the inner dynein arm of cilialy axonemes, and axonemal dyneins are molecular motors that drive the beating of cilia and flagella. Plotnikova *et al*. [47] have reported that loss of cilia in cancer cells may contribute to the insensitivity of cancer cells to environmental repressive signals, partly owing to derangement of cell cycle checkpoints governed by cilia and centrosomes. On the other hand, IPA analysis showed several genes interacting with *SRC* or *MYC* (Figure S6), each of which was reported as a representative gene in oncogenic pathways of ovarian cancer [25], [27]. Dressman *et al*. [25] have demonstrated that Src pathway activity is associated with chemotherapy response because of a significant correlation between the activation of Src pathway and poor prognosis in patients with platinum-resistant ovarian cancer. *MYC* is a multifunctional proto-oncogene and activated in about 30% of ovarian cancer by several mechanisms [48]. Iba *et al*. [49] report that MYC expression is associated with responsiveness to platinum-based chemotherapy and with prognosis in patients with epithelial ovarian cancer. Our PFS-related profile might have potentially functional relevance to altered activities of several oncogenic pathways. Although we identified several genes whose molecular function could be linked to prognosis in ovarian cancer patients, further functional study will be necessary to clarify the biological and pathological implications of the PFS-related profile.

These results suggest that the gene expression profile could be a useful tool to predict disease progression or recurrence of advanced-stage serous ovarian cancer. To apply the gene expression profile in clinical practice, we will need to improve the predictive ability of the profile and confirm the reliability of survival profile in a prospective multi-center study. Nevertheless, the survival-related profile could provide an optimization of the clinical management and development of new therapeutic strategies for the serous ovarian cancer patients.

## Materials and Methods

### Tissue Samples

One hundred ten Japanese patients who were diagnosed with advanced-stage serous ovarian cancer between July 1997 and June 2008 were included in this study. Fresh-frozen samples were obtained from primary tumor tissues during primary debulking surgery prior to chemotherapy. All patients with advanced-stage serous ovarian cancer were treated with platinum/taxane-based chemotherapy after surgery. In principle, patients were seen every 1 to 3 months for the first 2 years. Thereafter, follow-up visits had an interval of 3 to 6 months in the third to fifth year, and 6 to 12 months in the sixth to tenth year. At every follow-up visits, general physical and gynecologic examination were performed. CA125 serum levels were routinely determined. Staging of the disease was assessed according to the criteria of the International Federation of Gynecology and Obstetrics (FIGO) [19]. Optimal debulking surgery was defined as ≤1cm of gross residual disease. The histological characteristics of surgically resected specimens were assessed on formalin-fixed and paraffin-embedded hematoxylin and eosin sections by two or three gynecological pathologists belonging to the Japanese Society of Pathology at each institute, and frozen tissues containing more than 80% of tumor cells upon histological evaluation were used for RNA extraction. In this study, the degree of histological differentiation is determined according to the increase in the proportion of solid growth within the adenocarcinoma as follows: grade 1, less than 5% solid growth; grade 2, 6-50% solid growth; grade 3, over 50% solid growth based on grading system proposed by Japan Society of Gynecologic Oncology.

PFS time was calculated as the interval from primary surgery to disease progression or recurrence. Based on standard Response Evaluation Criteria In Solid Tumors (RECIST) guidelines [50], disease progression was defined as at least 20% increase in the sum of the longest diameters of all target lesions or as the appearance of one or more new lesions and/or unequivocal progression existing non-target lesions. Overall survival time was calculated as the interval from primary surgery to the death due to ovarian cancer. This study was approved by the institutional ethics review board at Niigata University (No. 239, 282, 285, and 318), Niigata Cancer Center Hospital (No. 25), Jichi Medical University (G07-01), Kagoshima City Hospital (H19-21), Hiroshima University (Hi-11), Nagasaki University (080509), Kumamoto University (No. 309), and Tokai University (07I-29). All patients provided written informed consent for the collection of samples and subsequent analysis.

### Microarray Experiments

Total RNA was extracted from tissue samples as previously described [17]. Five hundred nanograms of total RNA were converted into labeled cRNA with nucleotides coupled to a cyanine 3-CTP (Cy3) (PerkinElmer, Boston, MA, USA) using the Quick Amp Labeling Kit, one-color (Agilent Technologies). Cy3-labeled cRNA (1.65 µg) was hybridized for 17 hours at 65°C to an Agilent Whole Human Genome Oligo Microarray, which carries 60-mer probes to more than 40,000 human transcripts. The hybridized microarray was washed and then scanned in Cy3 channel with the Agilent DNA Microarray Scanner (model G2565AA). Signal intensity per spot was generated from the scanned image using Feature Extraction Software version 9.1 (Agilent Technologies) in the default settings. Spots that did not pass quality control procedures were flagged as “Absent”. The MIAME-compliant microarray data were deposited into the Gene Expression Omnibus data repository (accession number GSE17260).

### Microarray Data Analysis

We analyzed our dataset as a “discovery set” and the publicly available dataset as an “external dataset”. Considering differences in microarray platforms, we selected common genes between the Agilent Whole Human Genome Oligo Microarray and Affymetrix Human Genome U133 Plus 2.0 Array, which was the platform in an external dataset (GSE9891) [20].

Data normalization was performed in GeneSpring GX 10 (Agilent Technologies) as follows: (i) Threshold raw signals were set to 1.0. (ii) 75th percentile normalization was chosen as normalized algorithm. (iii) Baseline was transformed to median of all samples. Furthermore, the expression level was normalized by Z-transformation (the mean expression was set to 0 and standard deviation to 1 for each gene in each dataset). In our dataset, 18,178 probes with expression levels marked as “Present” in all microarrays were used to remove missing and uncertain signals on gene expression.

The PFS-related genes from the 18,178 probes were identified by univariate Cox proportional hazard analysis, followed by a ridge regression, a penalized Cox regression analysis for survival prediction (Figure S2). We first identified 97 probes with expression levels correlating with the PFS time determined using the univariate Cox proportional hazard model (*p*<0.01). In case of multiple probes representing a given gene (so-called multiple tagged gene) in microarrays, only the probe with the largest magnitude (i.e., sum of the squares of per-individual expression values) was extracted as a representative probe for the gene [24]. To avoid the problem of overfitting, ridge regression extension of the multivariate Cox model was employed [18]. The ridge regression shrinks regression coefficients (*β*) of genes in multivariate Cox model by imposing a penalty on squared values of the coefficients, and is able to handle the problem of having larger number of expression values than individuals in an appropriate way [30]. We estimated regression coefficients of the prognostic genes by the ridge regression Cox model using M-files (available at http://www.med.uio.no/imb/stat/bmms/software/microsurv/) for MATLAB (Mathworks, Natick, MA, USA). Using 10-fold cross-validation, we obtained regression coefficients with optimal penalty parameter for the penalized Cox model, and calculated a prognostic index for each patient as defined by
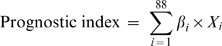
(1)where *β_i_* is the estimated regression coefficient of each gene in discovery dataset under ridge regression multivariate Cox model and *X_i_* is the Z-transformed expression value of each gene [18]. The estimated regression coefficient of each PFS-related gene given by ridge regression in the discovery set was also applied to calculate a prognostic index for each patient in external dataset using the equation above. We classified all patients into the two groups (high- and low-risk groups) by the median of the prognostic index in discovery set [9]. PFS between high- and low-risk groups was compared using Kaplan-Meier curves and the log rank test using GraphPad PRISM version 4.0 (GraphPad Software, San Diego, CA, USA). Furthermore, We then evaluated the prognostic index in the multivariate Cox proportional hazard model using JMP version 6 (SAS Institute, Cary, NC, USA). We also examined the discrimination performance of the prognostic index between early and late relapse in patients by plotting a receiver operating characteristic (ROC) curve for each dataset (JMP). Because 18 months is the median PFS time for advanced-stage ovarian cancer patients treated with cisplatin-paclitaxel [1], we used 18 months as the cut-off between early and late relapse. We performed ROC curve analysis for our prognostic index in only patients with follow-up for more than 18 months (Discovery set 103 samples; External dataset 84 samples).

To investigate the biological functions of PFS-related gene expression profiles, we used GO Ontology Browser, embedded in GeneSpring GX [17], [51]. The GO Ontology Browser was used to analyze which categories of gene ontology were statistically overrepresented among the gene list obtained. Statistical significance was determined by Fisher's exact test, followed by multiple testing corrections by the Benjamini and Hochberg false discovery rate (FDR) method [26]. Furthermore, we tried to explore molecular interaction networks among the PFS-related genes using Ingenuity Pathway Analysis (IPA) [17].

### Quantitative Real-Time Reverse Transcription Polymerase Chain Reaction (RT-PCR) Analysis

Real-time PCR was performed on *E2F2* (Hs00231667_m1, Applied Biosystems), *FOXJ1* (Hs00230964_m1, Applied Biosystems), *DNAH7* (Hs01022427_m1, Applied Biosystems), and *FILIP1* (Hs00325074_m1, Applied Biosystems) for a subset of serous ovarian cancer (n = 53) as previously described [17]. The relative quantification method [52] was used to measure the amounts of the respective genes in serous ovarian cancer samples, normalized to *ACTB* (Hs99999903_m1, Applied Biosystems) and *TBP* (Hs99999910_m1, Applied Biosystems).

### Evaluation of PFS-Related Genes in the External Dataset

To confirm whether our expression profile could predict prognosis of serous ovarian cancer patients in an independent data set, we selected to use publicly available microarray data (GSE9891) only because the data also disclosed individual clinical characteristics including PFS time. We examined clinical information of these dataset using supplementary data [20]. From this original dataset (n = 285), we selected 87 samples that were (i) diagnosed as advanced-stage serous adenocarcinoma, (ii) treated by platinum/taxane-based chemotherapy, (iii) obtained from primary lesion, and (iv) followed-up for more than 12 months (Table S1). Their samples are histologically graded by Silverberg classification [22] whose grading system is different from that in this study.

## Supporting Information

Figure S1 Kaplan-Meier survival curves between 110 patients in this dataset and 87 in Tothill's dataset.
(0.24 MB TIF)

Figure S2 Analytical process to develop a prognostic index for predicting survival.
(0.48 MB TIF)

Figure S3 Assessment of the sensitivity and specificity of 88-gene prognostic index using receiver-operating characteristic (ROC) curves. When early relapse is positive in the analysis, the area under ROC curve to distinguish early-relapse patients with less than 18 months of progression-free survival times from late-relapse patients was 0.959 and 0.674 in (A) discovery set (early, n = 54; late, n = 49) and in (B) external set (early, n = 45; late, n = 39), respectively.
(0.42 MB TIF)

Figure S4 Appling PFS-related gene expression profile to Dressman's dataset [25]. (A) Multivariate analysis showed a significant association of overall survival with the prognostic index estimated using the 88-gene linear combination model with the ridge regression coefficients from the present discovery set in Dresssman's dataset (HR, 1.51; 95% CI, 1.19–1.93, p = 0.0008) (B) Kaplan-Meier survival curves and the log rank test showed that high-risk patients had shorter overall survival compared to low-risk patients (median survival, 31 and 87 months for high- and low-risk patients, respectively; p = 0.0008).
(0.23 MB TIF)

Figure S5 Molecular interaction networks of 88 progression-free survival-related genes using Ingenuity Pathway Analysis (IPA) software. The prognostic genes incorporated into the respective networks were marked as gray-colored.
(2.42 MB TIF)

Figure S6 Molecular interaction networks of 88 progression-free survival-related genes using Ingenuity Pathway Analysis (IPA) software. The prognostic genes incorporated into the respective networks were marked as gray-colored.
(1.68 MB TIF)

Figure S7 Molecular interaction networks of 88 progression-free survival-related genes using Ingenuity Pathway Analysis (IPA) software. The prognostic genes incorporated into the respective networks were marked as gray-colored.
(1.82 MB TIF)

Table S1 Clinical characteristics of advanced-stage serous ovarian cancer patients in Tothill's dataset [20] (n = 87).
(0.04 MB DOC)

Table S2 Univariate and multivariate Cox's proportional hazard model analysis of prognostic factors for progression-free survival.
(0.04 MB DOC)

Table S3 Univariate Cox's proportional hazard model analysis of prognostic index for progression-free survival in the two datasets.
(0.04 MB DOC)

## References

[1] McGuire WP, Hoskins WJ, Brady MF, Kucera PR, Partridge EE (1996). Cyclophosphamide and cisplatin compared with paclitaxel and cisplatin in patients with stage III and stage IV ovarian cancer.. N Engl J Med.

[2] Piccart MJ, Bertelsen K, James K, Cassidy J, Mangioni C (2000). Randomized intergroup trial of cisplatin-paclitaxel versus cisplatin-cyclophosphamide in women with advanced epithelial ovarian cancer: three-year results.. J Natl Cancer Inst.

[3] Cannistra SA (2004). Cancer of the ovary.. N Engl J Med.

[4] du Bois A, Reuss A, Pujade-Lauraine E, Harter P, Ray-Coquard I (2009). Role of surgical outcome as prognostic factor in advanced epithelial ovarian cancer: a combined exploratory analysis of 3 prospectively randomized phase 3 multicenter trials: by the Arbeitsgemeinschaft Gynaekologische Onkologie Studiengruppe Ovarialkarzinom (AGO-OVAR) and the Groupe d'Investigateurs Nationaux Pour les Etudes des Cancers de l'Ovaire (GINECO).. Cancer.

[5] Winter WE, Maxwell GL, Tian C, Carlson JW, Ozols RF (2007). Prognostic factors for stage III epithelial ovarian cancer: a Gynecologic Oncology Group Study.. J Clin Oncol.

[6] Konstantinopoulos PA, Spentzos D, Cannistra SA (2008). Gene-expression profiling in epithelial ovarian cancer.. Nat Clin Pract Oncol.

[7] van 't Veer LJ, Dai H, van de Vijver MJ, He YD, Hart AA (2002). Gene expression profiling predicts clinical outcome of breast cancer.. Nature.

[8] Motoori M, Takemasa I, Yano M, Saito S, Miyata H (2005). Prediction of recurrence in advanced gastric cancer patients after curative resection by gene expression profiling.. Int J Cancer.

[9] Chen HY, Yu SL, Chen CH, Chang GC, Chen CY (2007). A five-gene signature and clinical outcome in non-small-cell lung cancer.. N Engl J Med.

[10] Schramm A, Schulte JH, Klein-Hitpass L, Havers W, Sieverts H (2005). Prediction of clinical outcome and biological characterization of neuroblastoma by expression profiling.. Oncogene.

[11] Bonome T, Levine DA, Shih J, Randonovich M, Pise-Masison CA (2008). A gene signature predicting for survival in suboptimally debulked patients with ovarian cancer.. Cancer Res.

[12] Crijns AP, Fehrmann RS, de Jong S, Gerbens F, Meersma GJ (2009). Survival-related profile, pathways, and transcription factors in ovarian cancer.. PLoS Med.

[13] Denkert C, Budczies J, Darb-Esfahani S, Györffy B, Sehouli J (2009). A prognostic gene expression index in ovarian cancer - validation across different independent data sets.. J Pathol.

[14] Hartmann LC, Lu KH, Linette GP, Cliby WA, Kalli KR (2005). Gene expression profiles predict early relapse in ovarian cancer after platinum-paclitaxel chemotherapy.. Clin Cancer Res.

[15] Spentzos D, Levine DA, Ramoni MF, Joseph M, Gu X (2004). Gene expression signature with independent prognostic significance in epithelial ovarian cancer.. J Clin Oncol.

[16] Agarwal R, Kaye SB (2006). Expression profiling and individualization of treatment for ovarian cancer.. Curr Opin Pharmacol.

[17] Yoshihara K, Tajima A, Komata D, Yamamoto T, Kodama S (2009). Gene expression profiling of advanced-stage serous ovarian cancers distinguishes novel subclasses and implicates ZEB2 in tumor progression and prognosis.. Cancer Sci.

[18] Bøvelstad HM, Nygård S, Størvold HL, Aldrin M, Borgan Ø (2007). Predicting survival from microarray data–a comparative study.. Bioinformatics.

[19] FIGO Cancer Committee. (1986). Staging Announcement: FIGO Cancer Committee.. Gynecol Oncol.

[20] Tothill RW, Tinker AV, George J, Brown R, Fox SB (2008). Novel molecular subtypes of serous and endometrioid ovarian cancer linked to clinical outcome.. Clin Cancer Res.

[21] International Federation of Gynecology and Obstetrics (1971). Classification and staging of malignant tumours in the female pelvis.. Acta Obstet Gynecol Scand.

[22] Silverberg SG (2000). Histopathologic grading of ovarian carcinoma: a review and proposal.. Int J Gynecol Pathol.

[23] Kommoss S, Schmidt D, Kommoss F, Hedderich J, Harter P (2009). Histological grading in a large series of advanced stage ovarian carcinomas by three widely used grading systems: consistent lack of prognostic significance. A translational research subprotocol of a prospective randomized phase III study (AGO-OVAR 3 protocol).. Virchows Arch.

[24] Woo HG, Park ES, Cheon JH, Kim JH, Lee JS (2008). Gene expression-based recurrence prediction of hepatitis B virus-related human hepatocellular carcinoma.. Clin Cancer Res.

[25] Dressman HK, Berchuck A, Chan G, Zhai J, Bild A (2007). An integrated genomic-based approach to individualized treatment of patients with advanced-stage ovarian cancer.. J Clin Oncol.

[26] Benjamini Y, Hochberg Y (1995). Controlling the false discovery rate: a practical and powerful approach to multiple testing.. J R Statist Soc B.

[27] Bild AH, Yao G, Chang JT, Wang Q, Potti A (2006). Oncogenic pathway signatures in human cancers as a guide to targeted therapies.. Nature.

[28] Dupuy A, Simon RM (2007). Critical review of published microarray studies for cancer outcome and guidelines on statistical analysis and reporting.. J Natl Cancer Inst.

[29] Bair E, Tibshirani R (2004). Semi-supervised methods to predict patient survival from gene expression data.. PLoS Biol.

[30] van Houwelingen HC, Bruinsma T, Hart AA, Van't Veer LJ, Wessels LF (2006). Cross-validated Cox regression on microarray gene expression data.. Stat Med.

[31] Rosenwald A, Wright G, Chan WC, Connors JM, Campo E (2002). The use of molecular profiling to predict survival after chemotherapy for diffuse large-B-cell lymphoma.. N Engl J Med.

[32] Sorlie T, Tibshirani R, Parker J, Hastie T, Marron JS (2003). Repeated observation of breast tumor subtypes in independent gene expression data sets.. Proc Natl Acad Sci U S A.

[33] Tawassoli FA, Devilee P (2003). Pathology and Genetics. Tumours of the Breast and Female Genital Organs..

[34] Malpica A, Deavers MT, Lu K, Bodurka DC, Atkinson EN (2004). Grading ovarian serous carcinoma using a two-tier system.. Am J Surg Pathol.

[35] Goodman MT, Howe HL, Tung KH, Hotes J, Miller BA (2003). Incidence of ovarian cancer by race and ethnicity in the United States, 1992–1997.. Cancer.

[36] McGuire V, Jesser CA, Whittemore AS (2002). Survival among U.S. women with invasive epithelial ovarian cancer.. Gynecol Oncol.

[37] Song H, Ramus SJ, Tyrer J, Bolton KL, Gentry-Maharaj A (2009). A genome-wide association study identifies a new ovarian cancer susceptibility locus on 9p22.2.. Nat Genet.

[38] Ozols RF (2005). Treatment goals in ovarian cancer.. Int J Gynecol Cancer.

[39] Katsumata N, Yasuda M, Takahashi F, Isonishi S, Jobo T (2009). Dose-dense paclitaxel once a week in combination with carboplatin every 3 weeks for advanced ovarian cancer: a phase 3, open-label, randomised controlled trial.. Lancet.

[40] Berchuck A, Iversen ES, Luo J, Clarke JP, Horne H (2009). Microarray analysis of early stage serous ovarian cancers shows profiles predictive of favorable outcome.. Clin Cancer Res.

[41] Helleman J, Jansen MP, Span PN, van Staveren IL, Massuger LF (2006). Molecular profiling of platinum resistant ovarian cancer.. Int J Cancer.

[42] Reimer D, Sadr S, Wiedemair A, Stadlmann S, Concin N (2007). Clinical relevance of E2F family members in ovarian cancer–an evaluation in a training set of 77 patients.. Clin Cancer Res.

[43] Callahan MJ, Nagymanyoki Z, Bonome T, Johnson ME, Litkouhi B (2008). Increased HLA-DMB expression in the tumor epithelium is associated with increased CTL infiltration and improved prognosis in advanced-stage serous ovarian cancer.. Clin Cancer Res.

[44] Lopez I, Mak EC, Ding J, Hamm HE, Lomasney JW (2001). A novel bifunctional phospholipase c that is regulated by Galpha 12 and stimulates the Ras/mitogen-activated protein kinase pathway.. J Biol Chem.

[45] Behrens P, Brinkmann U, Wellmann A (2003). CSE1L/CAS: its role in proliferation and apoptosis.. Apoptosis.

[46] Tanaka T, Ohkubo S, Tatsuno I, Prives C (2007). hCAS/CSE1L associates with chromatin and regulates expression of select p53 target genes.. Cell.

[47] Plotnikova OV, Golemis EA, Pugacheva EN (2008). Cell cycle-dependent ciliogenesis and cancer.. Cancer Res.

[48] Darcy KM, Brady WE, Blancato JK, Dickson RB, Hoskins WJ (2009). Prognostic relevance of c-MYC gene amplification and polysomy for chromosome 8 in suboptimally-resected, advanced stage epithelial ovarian cancers: a Gynecologic Oncology Group study.. Gynecol Oncol.

[49] Iba T, Kigawa J, Kanamori Y, Itamochi H, Oishi T (2004). Expression of the c-myc gene as a predictor of chemotherapy response and a prognostic factor in patients with ovarian cancer.. Cancer Sci.

[50] Therasse P, Arbuck SG, Eisenhauer EA, Wanders J, Kaplan RS (2000). New guidelines to evaluate the response to treatment in solid tumors. European Organization for Research and Treatment of Cancer, National Cancer Institute of the United States, National Cancer Institute of Canada.. J Natl Cancer Inst.

[51] Okada H, Tajima A, Shichiri K, Tanaka A, Tanaka K (2008). Genome-wide expression of azoospermia testes demonstrates a specific profile and implicates ART3 in genetic susceptibility.. PLoS Genet.

[52] Livak KJ, Schmittgen TD (2001). Analysis of Relative Gene Expression Data Using Real-Time Quantitative PCR and the 2−ΔΔCT Method.. Methods.

