# Supplementary figures and images for: Gene Expression Profile for Predicting Survival in Advanced-Stage Serous Ovarian Cancer Across Two Independent Datasets
Source: PLoS One. 2010 Mar 12;5(3):e9615. doi: 10.1371/journal.pone.0009615 (PMC2837379; doi:10.1371/journal.pone.0009615)

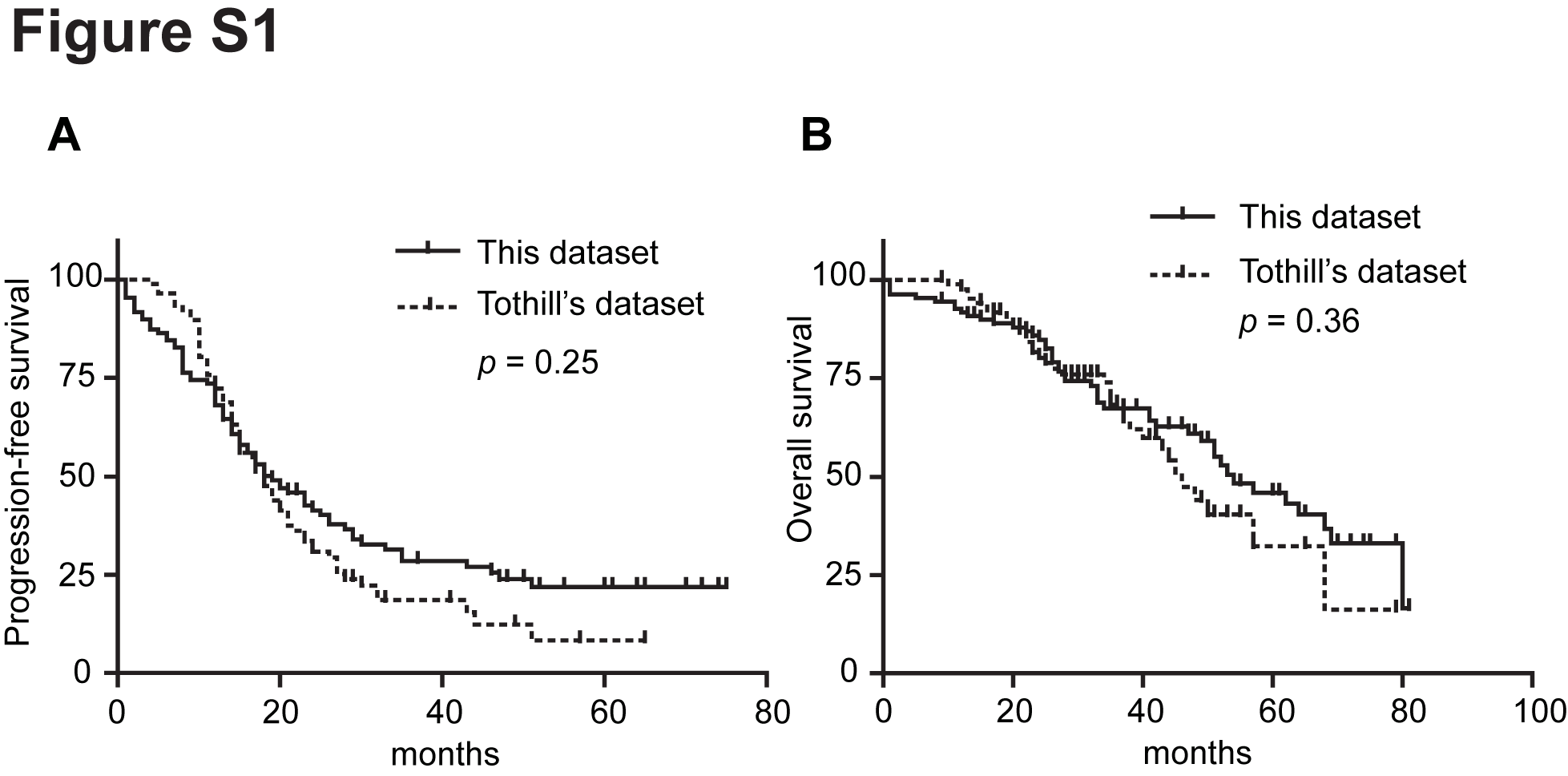

Supplement: Figure S1 — Kaplan-Meier survival curves between 110 patients in this dataset and 87 in Tothill's dataset. (0.24 MB TIF) [file pone.0009615.s001.tif]

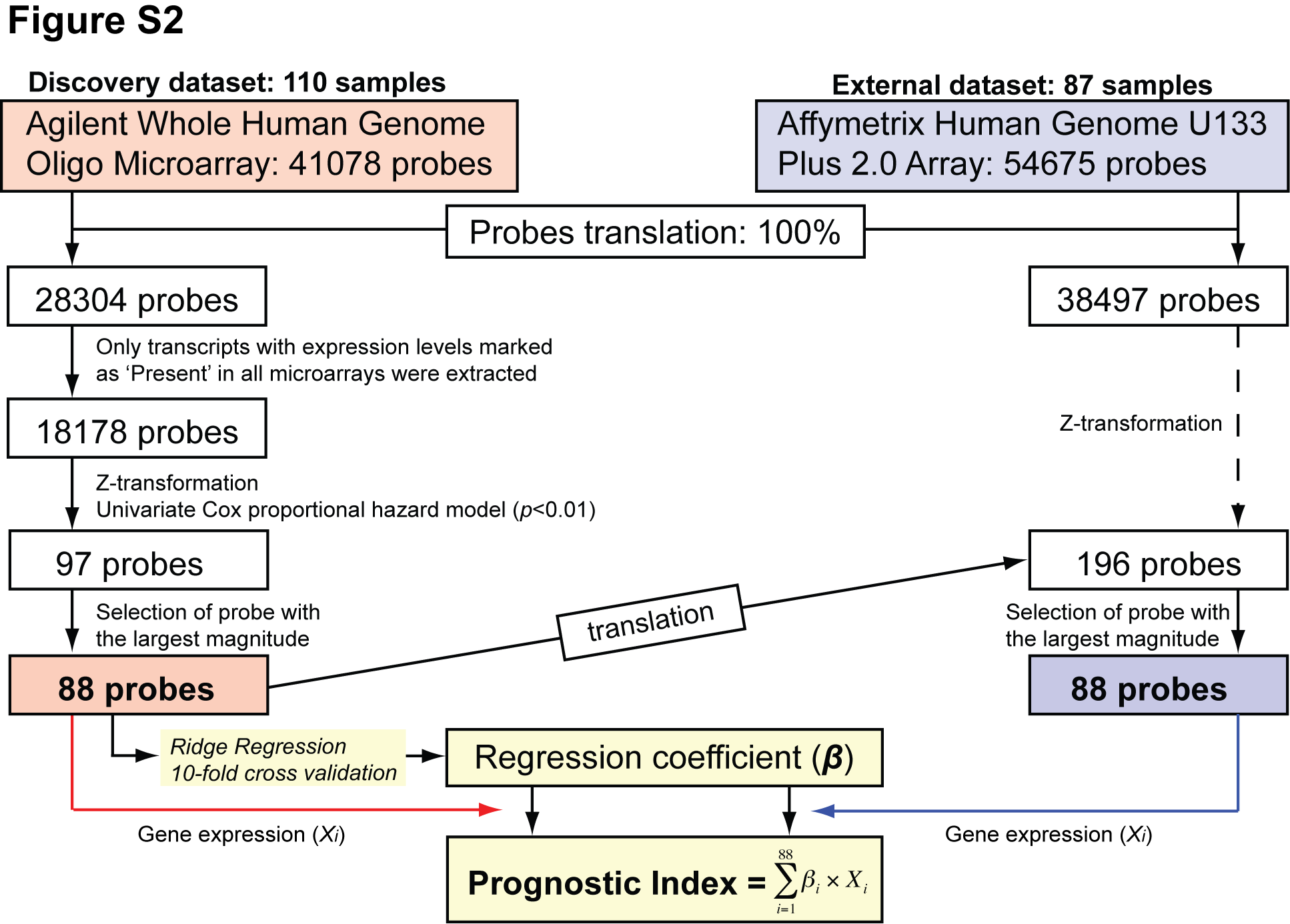

Supplement: Figure S2 — Analytical process to develop a prognostic index for predicting survival. (0.48 MB TIF) [file pone.0009615.s002.tif]

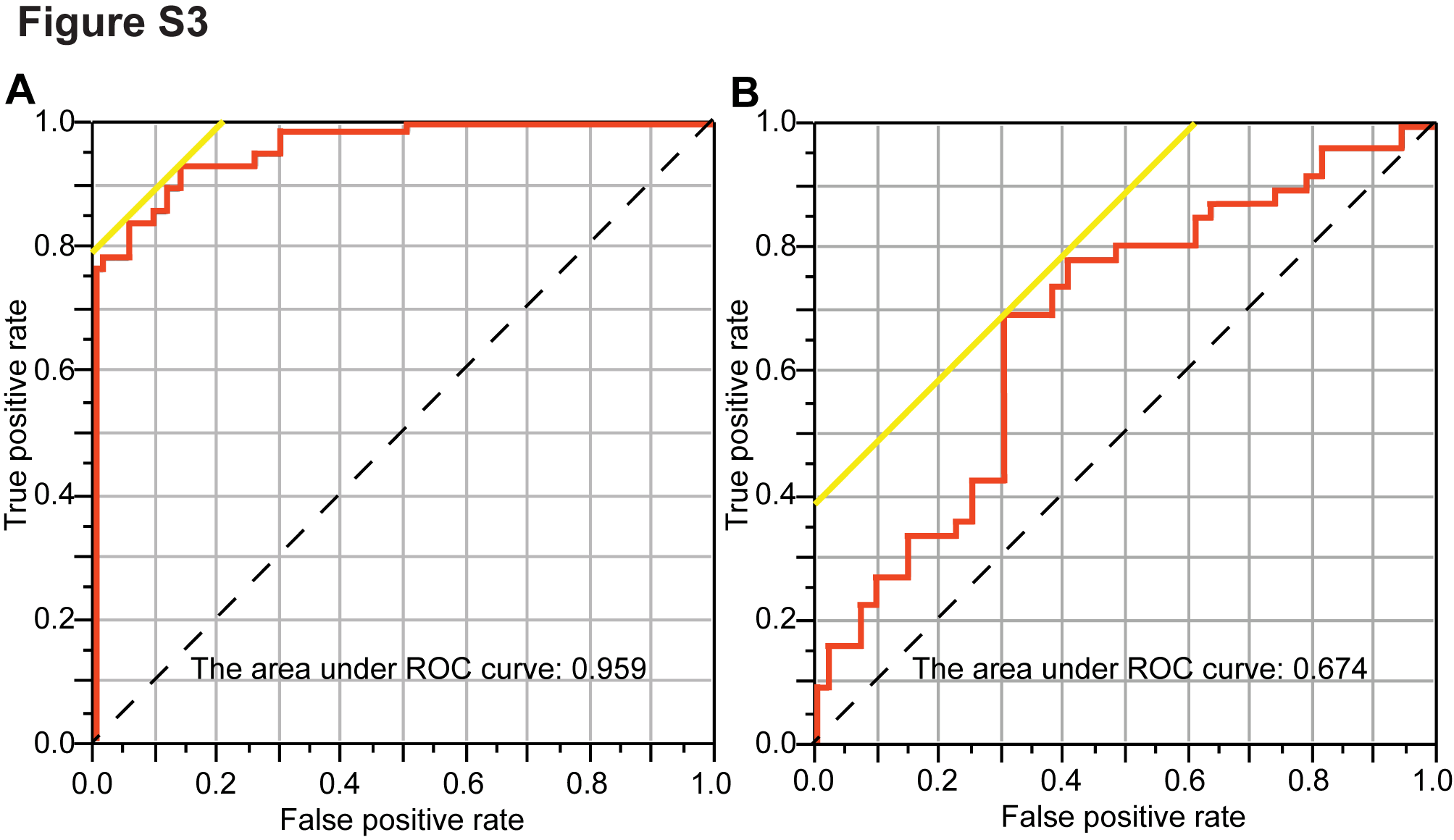

Supplement: Figure S3 — Assessment of the sensitivity and specificity of 88-gene prognostic index using receiver-operating characteristic (ROC) curves. When early relapse is positive in the analysis, the area under ROC curve to distinguish early-relapse patients with less than 18 months of progression-free survival times from late-relapse patients was 0.959 and 0.674 in (A) discovery set (early, n = 54; late, n = 49) and in (B) external set (early, n = 45; late, n = 39), respectively. (0.42 MB TIF) [file pone.0009615.s003.tif]

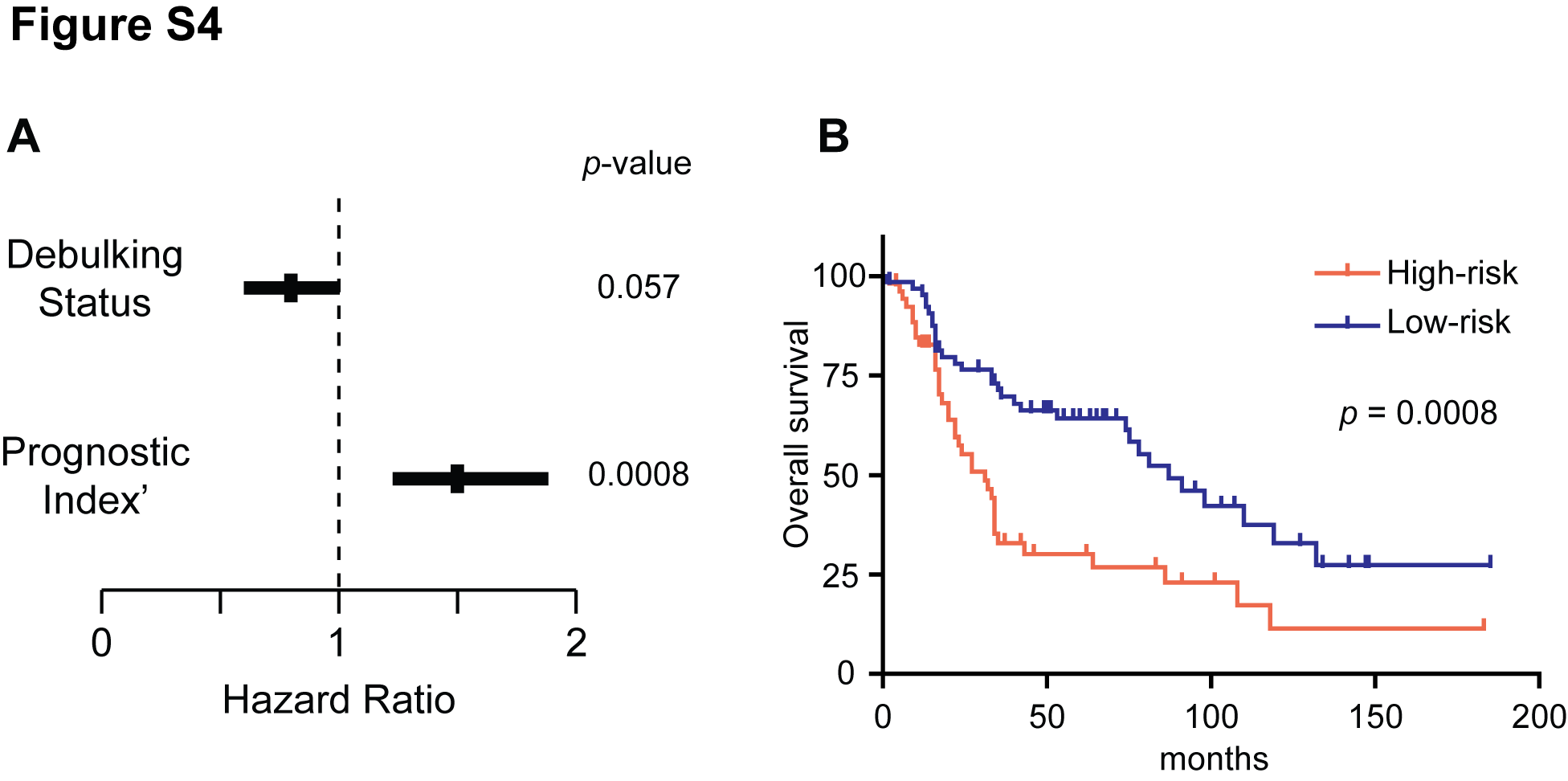

Supplement: Figure S4 — Appling PFS-related gene expression profile to Dressman's dataset [25]. (A) Multivariate analysis showed a significant association of overall survival with the prognostic index estimated using the 88-gene linear combination model with the ridge regression coefficients from the present discovery set in Dresssman's dataset (HR, 1.51; 95% CI, 1.19–1.93, p = 0.0008) (B) Kaplan-Meier survival curves and the log rank test showed that high-risk patients had shorter overall survival compared to low-risk patients (median survival, 31 and 87 months for high- and low-risk patients, respectively; p = 0.0008). (0.23 MB TIF) [file pone.0009615.s004.tif]

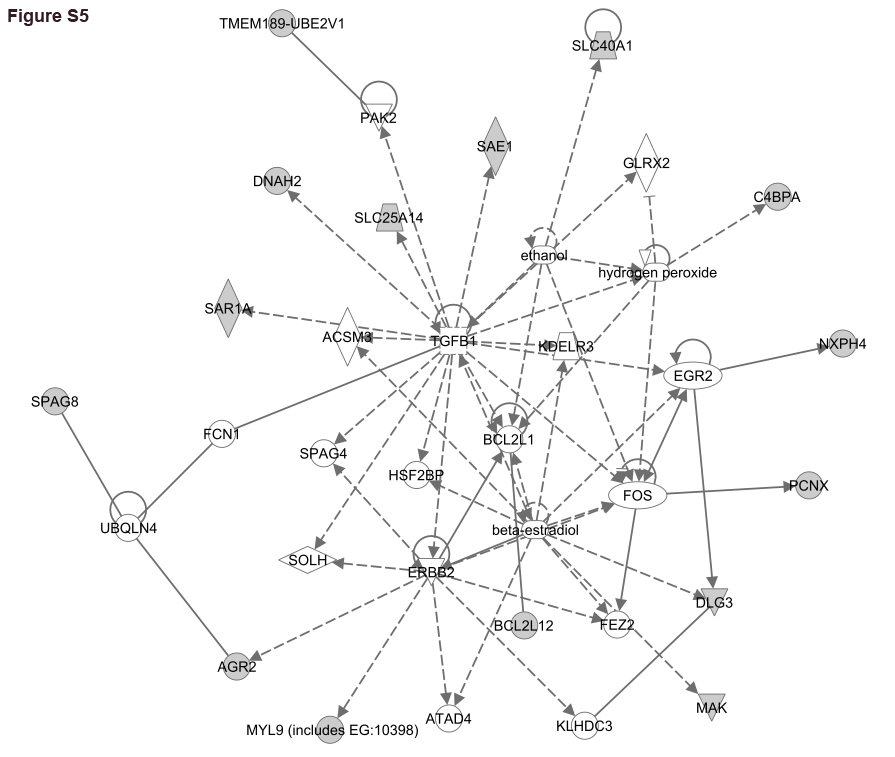

Supplement: Figure S5 — Molecular interaction networks of 88 progression-free survival-related genes using Ingenuity Pathway Analysis (IPA) software. The prognostic genes incorporated into the respective networks were marked as gray-colored. (2.42 MB TIF) [file pone.0009615.s005.tif]

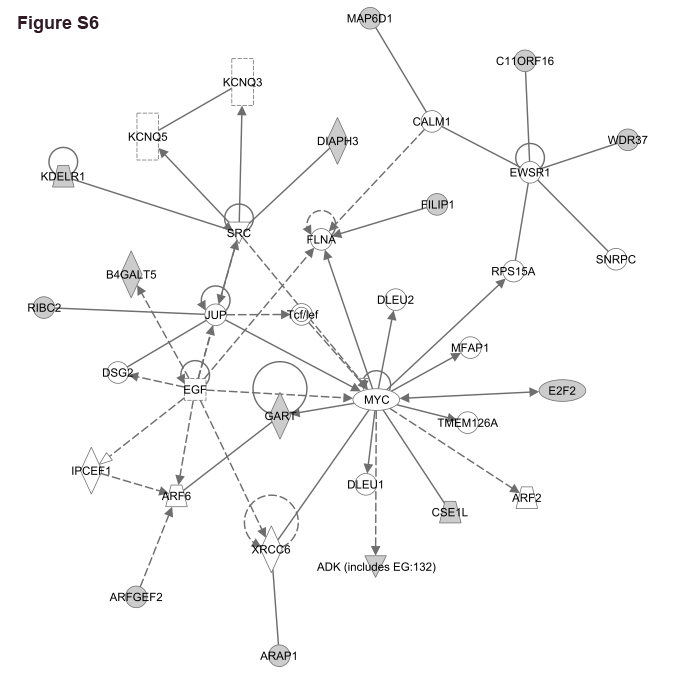

Supplement: Figure S6 — Molecular interaction networks of 88 progression-free survival-related genes using Ingenuity Pathway Analysis (IPA) software. The prognostic genes incorporated into the respective networks were marked as gray-colored. (1.68 MB TIF) [file pone.0009615.s006.tif]

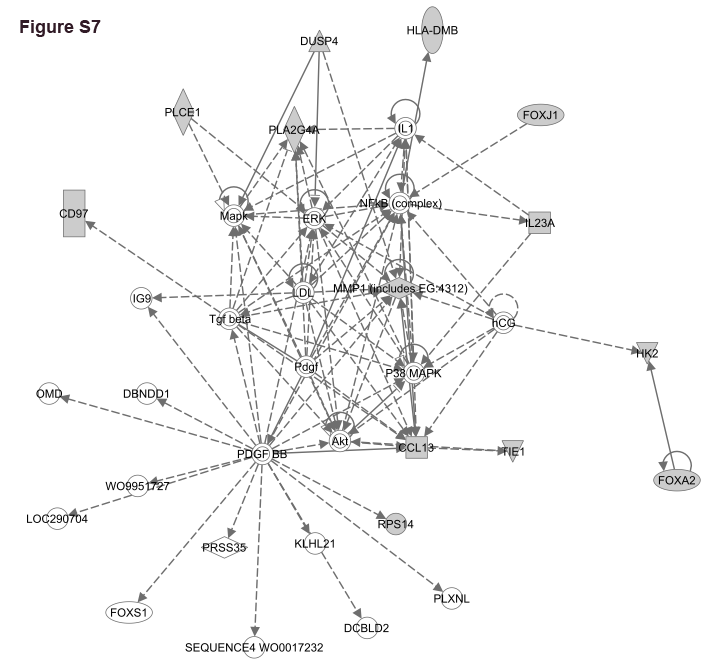

Supplement: Figure S7 — Molecular interaction networks of 88 progression-free survival-related genes using Ingenuity Pathway Analysis (IPA) software. The prognostic genes incorporated into the respective networks were marked as gray-colored. (1.82 MB TIF) [file pone.0009615.s007.tif]
