# Supplementary material for: Gene Expression Profile for Predicting Survival in Advanced-Stage Serous Ovarian Cancer Across Two Independent Datasets
Source: PLoS One. 2010 Mar 12;5(3):e9615. doi: 10.1371/journal.pone.0009615 (PMC2837379; doi:10.1371/journal.pone.0009615)
Supplement: Table S1 — Clinical characteristics of advanced-stage serous ovarian cancer patients in Tothill's dataset [20] (n = 87). (0.04 MB DOC) [file pone.0009615.s008.doc]

Table S1

Comparison of clinical characteristics of advanced-stage serous ovarian cancer patients in this dataset and Tothill’s dataset [20]

|  | This dataset | Tothill's dataset | *p*-value |
| --- | --- | --- | --- |
|  | n = 110 | n = 87 |  |
| Age | 59.0±12.6 | 59.2±9.5 | 0.90† |
|  |  |  |  |
| Stage |  |  | 0.0015‡ |
| stage III | 93 | 85 |  |
| stage IV | 17 | 2 |  |
|  |  |  |  |
| Debulking Surgery |  |  | 0.19‡ |
| optimal | 57 | 54 |  |
| not optimal | 53 | 33 |  |
|  |  |  |  |
| Grade | Three-tier systems proposed by JSOG* | Silverberg classification | - |
| grade 1 | 26 | 0 |  |
| grade 2 | 42 | 30 |  |
| grade 3 | 42 | 57 |  |

Differences in clinical characteristics between this dataset and Tothill's dataset were tested using the †unpaired t-test, and ‡Fisher’s exact test. *JSOG denotes Japan Society of Gynecologic Oncology.
