# Supplementary material for: Gene Expression Profile for Predicting Survival in Advanced-Stage Serous Ovarian Cancer Across Two Independent Datasets
Source: PLoS One. 2010 Mar 12;5(3):e9615. doi: 10.1371/journal.pone.0009615 (PMC2837379; doi:10.1371/journal.pone.0009615)
Supplement: Table S2 — Univariate and multivariate Cox's proportional hazard model analysis of prognostic factors for progression-free survival. (0.04 MB DOC) [file pone.0009615.s009.doc]

**Table S2**

**Univariate and multivariate Cox’s proportional hazard model analysis of prognostic factors for progression-free survival**

1) Present study (n = 110)

|  | Univariate analysis |  |  | Multivariate analysis |  |
| --- | --- | --- | --- | --- | --- |
| Prognostic factor | Hazard ratio (95%CI*) | *p*-value |  | Hazard ratio (95%CI) | *p*-value |
| **Age** | 0.99 (0.97-1.01) | 0.41 |  | 1.00 (0.98-1.02) | 0.93 |
| **Stage IV (vs Stage III)** | 1.40 (1.05-1.81) | 0.022 |  | 1.25 (0.93-1.64) | 0.14 |
| **Optimal Surgery (vs not optimal)** | 0.57 (0.45-0.72) | <0.0001 |  | 0.73 (0.56-0.94) | <0.0001 |
| **Grade** |  |  |  |  |  |
| **Grade2 (vs Grade1)** | 1.21 (0.89-1.67) | 0.23 |  | 1.06 (0.77-1.48) | 0.74 |
| **Grade3 (vs Grade1)** | 1.44 (1.07-1.98) | 0.016 |  | 1.18 (0.86-1.65) | 0.3 |

2) Tothill’s study (n = 87)

|  | Univariate analysis |  |  | Multivariate analysis |  |
| --- | --- | --- | --- | --- | --- |
| Prognostic factor | Hazard ratio (95%CI) | *p*-value |  | Hazard ratio (95%CI) | *p*-value |
| **Age** | 1.01 (0.98-1.03) | 0.61 |  | 1.01 (0.98-1.04) | 0.51 |
| **Stage IV (vs Stage III)** | 1.26 (0.51-2.28) | 0.55 |  | 1.07 (0.43-1.97) | 0.60 |
| **Optimal Surgery (vs not optimal)** | 0.78 (0.62-0.99) | 0.049 |  | 0.78 (0.61-1.00) | 0.053 |

*CI denotes confidence interval.
