# Supplementary material for: Gene Expression Profile for Predicting Survival in Advanced-Stage Serous Ovarian Cancer Across Two Independent Datasets
Source: PLoS One. 2010 Mar 12;5(3):e9615. doi: 10.1371/journal.pone.0009615 (PMC2837379; doi:10.1371/journal.pone.0009615)
Supplement: Table S3 — Univariate Cox's proportional hazard model analysis of prognostic index for progression-free survival in the two datasets. (0.04 MB DOC) [file pone.0009615.s010.doc]

**Table S3**

**Univariate Cox’s proportional hazard model analysis of prognostic index for progression-free survival in the two datasets.**

|  | This dataset | Tothill's dataset |
| --- | --- | --- |
| **1) stage III, G2/3, optimal surgery** | **n = 35** | **n = 54** |
| 175 PFS-genes expression profile **(p<0.01)** |  |  |
| HR (vs low) | 5.47 | 1.18 |
| 95% CI | - | 0.85-1.63 |
| *p*-value | *p* < 0.0001 | *p* = 0.32 |
|  |  |  |
| 17 PFS-genes expression profile **(p<0.001)** |  |  |
| HR (vs low) | 6.53 | 0.84 |
| 95% CI | 2.89-27.9 | 0.60-1.16 |
| *p*-value | *p* < 0.0001 | *p* = 0.30 |
|  |  |  |
| **2) stage III, G2/3, suboptimal surgery** | **n = 34** | **n = 31** |
| 122 PFS-genes expression profile **(p<0.01)** |  |  |
| HR (vs low) | 4.032 | 1.05 |
| 95% CI | - | 0.72-1.55 |
| *p*-value | *p* < 0.0001 | *p* = 0.79 |
|  |  |  |
| 5 PFS-genes expression profile **(p<0.001)** |  |  |
| HR (vs low) | 2.55 | 1.49 |
| 95% CI | 1.66-4.08 | 1.02-2.21 |
| *p*-value | *p* < 0.0001 | *p* = 0.042 |
